# Obesity: a disease of the *ponderostat* and the regulation of energy balance

**DOI:** 10.1007/s40519-025-01790-9

**Published:** 2025-10-15

**Authors:** Silvio Buscemi, Carola Buscemi, Piero Colombrita, Cristiana Randazzo, Anna Maria Barile, Giorgio Arnaldi

**Affiliations:** 1 Department of Promozione Della Salute, Materno-Infantile, Medicina Interna E Specialistica Di Eccellenza (PROMISE), University of Palermo, Palermo, Italy; 2 Clinical Nutrition, Obesity and Metabolic Diseases Unit, University Hospital Policlinico “P. Giaccone”, Edificio 2° - Piazza Delle Cliniche, 2, 90127 Palermo, Italy; 3 Internal Medicine Unit, “V. Cervello” Hospital, Ospedali Riuniti “Villa Sofia-Cervello”, Palermo, Italy; 4 Endocrinology Unit, University Hospital Policlinico “P. Giaccone”, Palermo, Italy

**Keywords:** Obesity, Energy balance, Hunger, Satiety, Energy expenditure, Ponderostat

## Abstract

Energy balance and thermodynamic laws regulate body weight. Therefore, obesity must occur over a sufficiently long period of time in which energy intake exceeds energy expenditure. It is clear that a strict application of the law of energy balance cannot fully explain what is observed in real life. One possible hypothesis is that some individuals may have an energy-sparing metabolism, predisposing them to obesity. Furthermore, energy balance can be regulated to maintain body weight within a fixed individual range, a set point, which is influenced by genetic or epigenetic factors. An energy balance regulator, the ponderostat, may be able to increase or decrease both energy expenditure and energy intake, depending on food availability (e.g., periods of famine or low-calorie diet, periods of overeating), to maintain body weight within the set point. The ponderostat is regulated by a complex neuroendocrine system that includes central structures located in the frontal cortex, hypothalamus, and diencephalic region, with peripheral afferents and efferents. Therefore, in many cases, obesity could be considered the consequence of a dysregulated ponderostat. This narrative review proposes a unifying perspective that considers obesity as a biological condition with an abnormal set point and dysregulated energy balance due to abnormalities in ponderostat function. Current and future antiobesity pharmacological treatments may be considered curative for ponderostat dysregulation.

## Introduction

Energy balance (EB) is a fundamental law of physics that regulates body weight and energy stores. According to the second principle of thermodynamics, energy is neither created nor destroyed but transformed into heat that is dispersed into the environment [1]. The EB describes the dynamic relationship between the energy intake (EI) derived from macronutrients (carbohydrates, lipids, proteins, and alcohol) and the energy used by the body to perform work (energy expenditure, EE). Assuming that the body is a thermodynamic machine, the energy balance equation, EI—EE = △ energy stores, is a simplified model that describes the relationships among the EI, EE, and changes in the body’s energy reserves. When EI equals EE (neutral EB), body weight remains stable, as energy intake is matched by the body’s metabolic demands, without the accumulation or generation of energy reserves. In contrast, a positive EB (EI > EE) leads to the storage of surplus energy in the form of adipose tissue, glycogen, and, to a lesser extent, protein, contributing to weight gain. A negative EB (EI < EE) induces the mobilization of energy reserves, resulting in weight loss. Therefore, obesity necessarily results from a sufficiently prolonged period in which the EB has remained positive. Obesity is a significant global public health problem, as its prevalence has been steadily increasing in recent decades [2]. This increasing trend may be related to both environmental (such as urbanization) and individual (genetic/epigenetic) factors. Urbanization has contributed to a progressive increase in the prevalence of obesity in the past century. According to WHO data from 2013, there has been an increase in residence in urban areas, and in 2010, more than 50% of people were reported to live in urban areas [3]. By 2050, more than 65% of the population is expected to reside in urban areas. This trend has been accompanied by a progressive reduction in daily physical activity and related energy expenditure [4]**.** Urbanization has also led to an increase in the use of ultraprocessed foods with a consequent progressive increase in habitual caloric intake [5]**.** Ultraprocessed food has an obesogenic nutritional profile because it tends to have high energy density, high free sugar and total fat content, and low fiber content. This food is also less satiating and often has a high glycemic load. In addition, it is aggressively marketed with large portions that are designed to be eaten as a snack. All of these factors promote excessive calorie consumption, which contributes to a positive EB and thus to overweight and obesity [6–8]. A simplistic conclusion is that obesity treatment can be summed up in the phrase "eat less and move more." Unfortunately, daily clinical experience confirms that obesity requires complex treatments and that energy balance often fails to be negatively affected for biological reasons. Although studies on energy metabolism have been available for many years, no reliable conclusions have been reached to date regarding the possibility that some energy-sparing individuals may be biologically predisposed to obesity. Recently, effective antiobesity pharmacological treatments have become available that are able to reduce the EI, and other drugs in the near future will soon also be able to increase the EE; thus, understanding the complex regulatory mechanisms of energy balance is important for obtaining more accurate diagnoses and more tailored treatments. The aim of this review is to provide a unified description of the regulatory mechanisms of energy balance and its clinical implications, taking a comprehensive view of the current knowledge.

## Energy expenditure

**Fig. 1:**
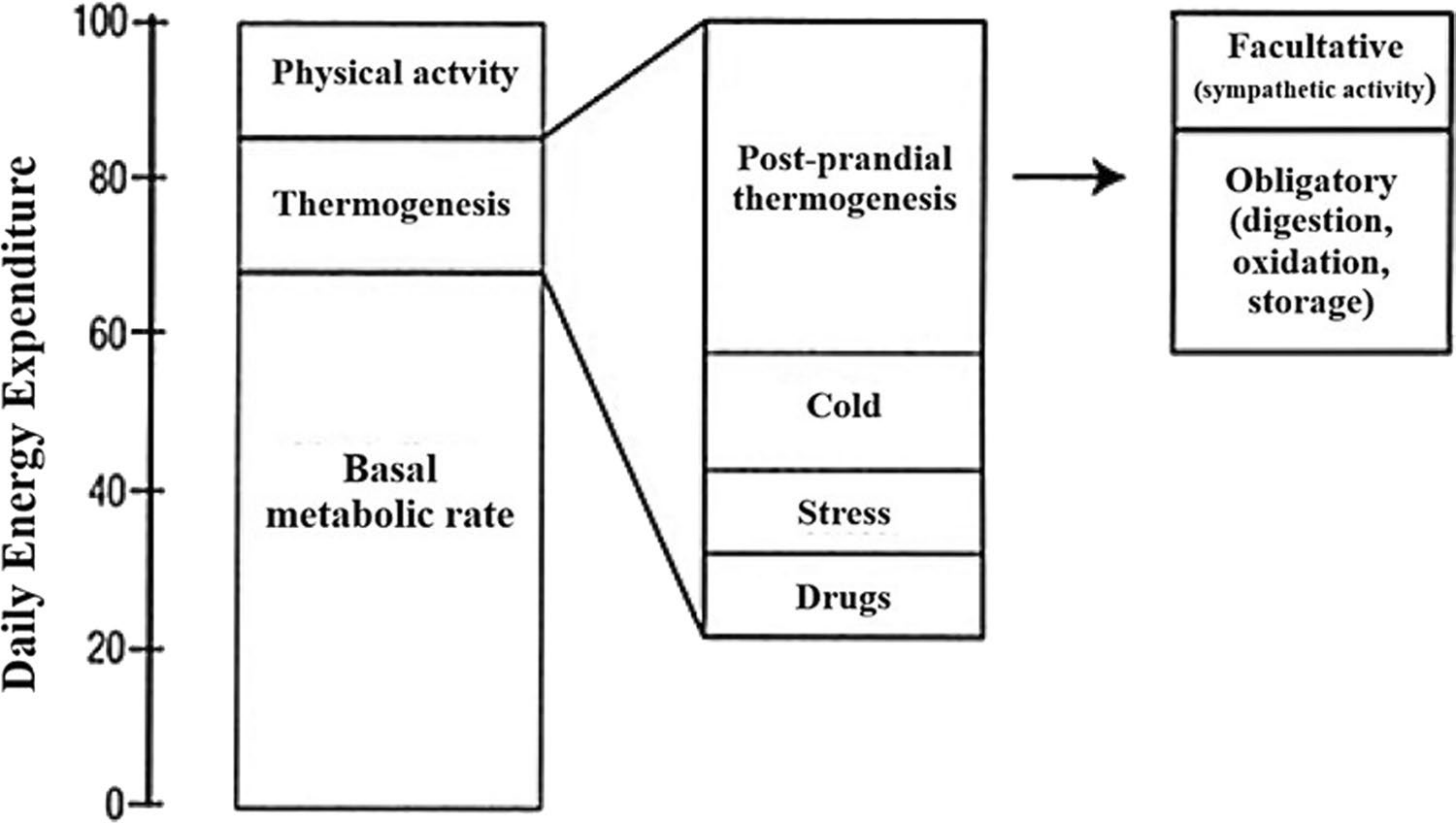
Schematic representation of the components of daily energy expenditure

Energy expenditure (EE) is the total amount of energy the body uses over a given time, typically 24 h, and consists of three main components: the basal metabolic rate (BMR), physical activity, and thermogenesis (Fig. 1).

**Fig. 2:**
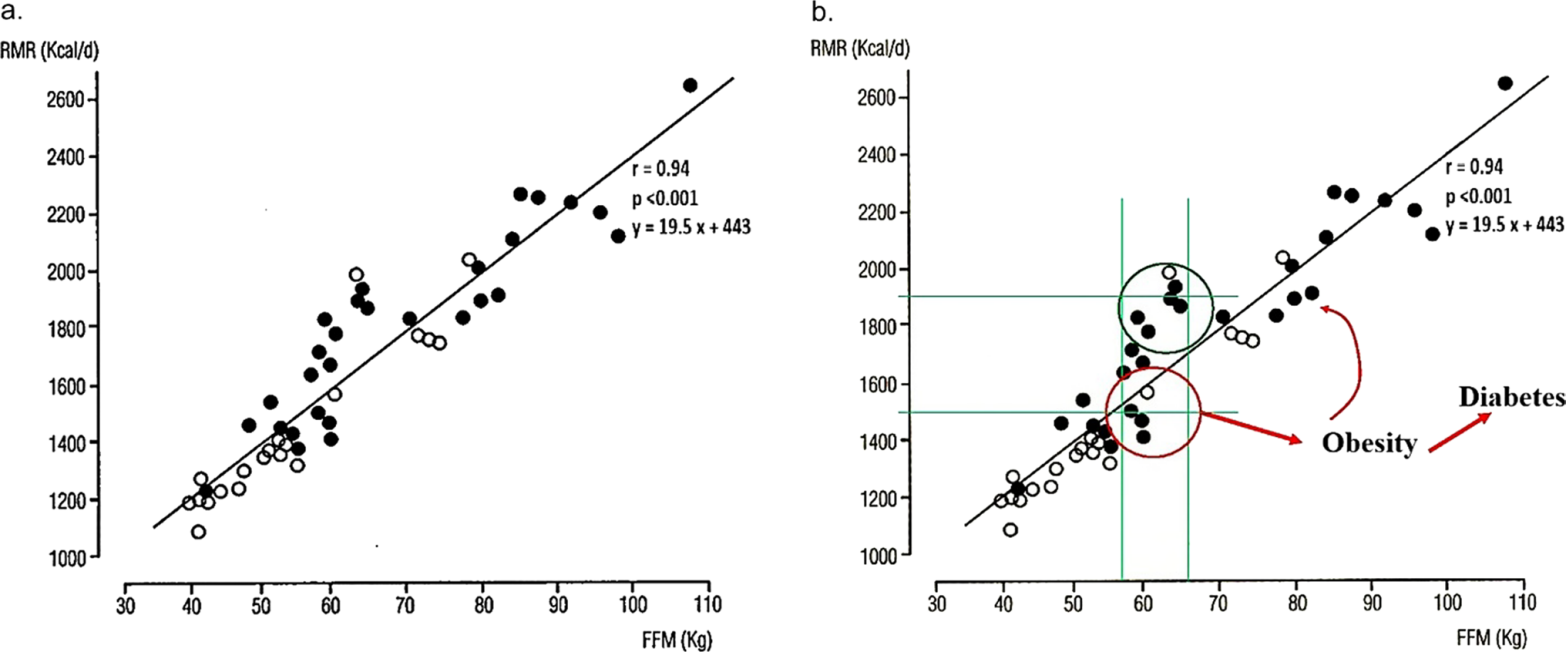
**a** Correlations between the resting metabolic rate (RMR) and fat-free mass (FFM). **b** Possible evidence of low relative RMR in preobese individuals (adapted from Ref. [9]). In particular, it is highlighted that a narrow range of FFM corresponds to a wide variability in RMR. Those individuals with lower RMR (inside the red circle) are more predisposed to developing obesity, with a consequent increase in FFM and thermogenic response. Consequently, once obesity establishes itself, the relative deficit in energy expenditure is no longer easily identifiable.

The BMR refers to the energy required to maintain essential physiological functions at rest under standardized conditions (postabsorptive fasting, a thermoneutral environment and complete physical rest) and is measured in the morning at bed soon after awakening. It accounts for approximately 60–70% of the total daily EE in adult, sedentary individuals. As the BMR is difficult to measure in the routine clinical setting, mainly because the individual to be tested needs to spend the night at the calorimetry laboratory, the resting metabolic rate (RMR) is often measured under the same conditions as the BMR, but the individual to be tested reaches the laboratory moving from home. The RMR is approximately 10% greater than the BMR. Several factors influence both BMR and RMR, including age, sex, hormonal status, fat-free mass (FFM) or body cell mass (BCM), genetic factors, and physiological or pathological conditions. FFM includes muscles, organs, bones, and body water and is the strongest determinant of BMR/RMR, as it includes the most metabolically active tissues, mainly muscle tissue (Fig. 2a) [9]. Compared with women, men typically have higher BMRs because of their greater muscle mass, whereas aging and sarcopenia contribute to a progressive decline in BMR. The decrease in BMR with aging may contribute to the weight gain that often occurs with advancing age. Compared with nonobese people, obese people have greater amounts of FFM, expressed in absolute value (kg); thus, the absolute value of the BMR/RMR (kcal/24 h) is greater in obese individuals than in nonobese individuals. Usually, to compare the BMR/RMR between people with different body sizes, to investigate the presence of metabolically efficient states, the energy expenditure needs to be normalized for the FFM (i.e., kcal/kg-FFM 24 h). Additionally, the RMR is inversely correlated with insulinemia and insulin resistance, a hallmark of people with central obesity–metabolic syndrome, and positively correlated with serum glucagon concentrations [10]. Physical activity represents the most variable component of EE, contributing approximately 15–30%, but it can vary widely depending on individual physical activity level. Regular physical activity can increase muscle mass, BMR and RMR [11]. Different aspects of physical activity must be considered, as it depends on work activity, habitual level of physical activity, intensity, duration, and type of physical activity. In physics, the work (*W*) to engage in physical activity is defined as the product of the force (*F*) applied to an object and the distance (*d*) the object moves in the direction of that force. The corresponding equation is *W* = *F* × *d*; according to Newton’s second law (*F* is equal to the mass (*m*) × acceleration (*a*)), it can be rewritten as *W* = (*m* × *a*) × *d*. Therefore, the EE increases with increasing mass, namely, body weight. Therefore, there is clear evidence that, for the same distance and acceleration, people with obesity spend more energy than normal-weight individuals do. Physical activity is an important component of obesity treatment, and even a simple walk results in significant EE. Unfortunately, people with obesity are often sedentary [12, 13], and their attitudes toward more active lifestyles need to be improved [14]. Thermogenesis is the production of heat by the body; it represents approximately 10% of the total EE but varies considerably between individuals. Its regulation may involve brown adipose tissue (BAT) and beige adipocytes, which generate heat through uncoupling protein-1 (UCP-1). BAT activity is highest in infants but subsequently decreases until adulthood, when it is expressed mainly in lateral-cervical, paravertebral, and supraclavicular sites. Similar to cells in BAT are beige adipocytes that may transdifferentiate from white adipocytes and back inside the adipose tissue (browning) [15]. This transition can be stimulated by cold exposure, sympathetic nervous system activity, certain medications, and the exercise-induced hormone irisin [16]. Thermogenesis includes other components, including postprandial thermogenesis (PPT), which consists of an increase in EE following the ingestion of a meal. Two components of PPT, obligatory and facultative thermogenesis, can be distinguished. The obligatory component of PPT consists of the energy cost of digestion, absorption, and nutrient storage. The facultative component of PPT is consequent to postprandial sympathetic nervous system (SNS) activation and may be prevented by the concomitant administration of beta-blockers. Other factors influencing PPT include meal size, body composition, age, habitual physical activity, and insulin sensitivity. Protein causes the highest PPT (20–30%), followed by carbohydrates (5–10%) and fats (0–3%). PPT is distinguished from *diet-induced thermogenesis* (DIT), which is related to the influence of nutritional status on EE; more specifically, under conditions of overnutrition (positive EB), the body tends to dissipate the extra caloric intake, increasing EE (the DIT increases), and undernutrition (negative EB) induces the opposite effect, thus reducing DIT. Stress-induced thermogenesis is driven by sympathetic activation and catecholamine release, which increase heart rate, lipolysis, and EE. For instance, patients with polytrauma or extensive burns experience stress-induced thermogenesis, and their BMR/RMR value increases by at least 10%. Environmental and behavioral factors also influence EE. Therefore, exposure to low temperatures can increase EE (cold-induced thermogenesis), as the body works to maintain homeostasis. Additionally, seasonal or geographic factors may influence the EE. Pharmacological thermogenesis is triggered by some substances, such as caffeine, nicotine, and ephedrine [17, 18]**.** In contrast, other drugs, such as beta-blockers, reduce sympathetic nervous system activity and may decrease EE. Conversely, overfeeding and some pharmacological agents that increase sympathetic nervous system activity (e.g., sympathomimetics, theophylline, salbutamol, and thyroxine at supraphysiological doses) can increase EE.

## Methods for investigating energy expenditure

Direct calorimetry (DC), which directly measures the heat produced by the body, is considered the gold standard for measuring EE. Nevertheless, DC is expensive, complex, and impractical for most centers. Therefore, indirect calorimetry (IC) is the most widely used technique for measuring EE in clinical practice. Validation studies with DC have demonstrated that IC is a reliable and accurate method for measuring EE; furthermore, IC may provide interesting information about the oxidation rate of substrates (lipids, carbohydrates, and proteins). Briefly, IC is a noninvasive technique that estimates EE by measuring oxygen consumption (VO_2_) and carbon dioxide production (VCO_2_). These values are used to calculate the respiratory quotient (RQ = VCO₂/VO₂), indicating the proportion of carbohydrates and fats oxidized. From VO₂ and RQ, it is possible to calculate [19] the oxygen caloric equivalent (the amount of energy released during the consumption of 1 L of oxygen according to the mixture of substrates oxidized) to obtain the EE value. Measuring EE (practically the BMR/RMR) and the substrate oxidation rate provides useful information about nutritional status, especially in patients with malnutrition or sarcopenia, and allows the investigation of causative factors of obesity involving the EB. It is also possible to estimate individual energy requirements according to precision medicine in the management of excess body weight, monitoring the response to diet and exercise, and investigating the effects of new antiobesity drugs. Interestingly, indirect calorimetry can be applied to the metabolic chamber. Compared to metabolic chambers using the direct calorimetry method, the metabolic chambers with indirect calorimetry system, while offering a good level of accuracy, have the advantage of being relatively less expensive and providing more information in terms of measuring daily energy expenditure and its components, as well as the oxidation of energy substrates. This information cannot be obtained using indirect calorimetry equipment solely using the ventilated hood system [20].

## Energy intake

Energy intake (EI) is the individual amount of energy introduced with macronutrients through food and drink. Each macronutrient provides a specific caloric value: carbohydrates and proteins supply approximately 4 kcal/g (17 kJ/g), fats approximately 9 kcal/g (38 kJ/g), and alcohol approximately 7 kcal/g (29 kJ/g). The most accurate method for measuring the caloric content of food is the calorimetric bomb, which consists of burning food samples to measure the heat released. However, this method is impractical for large scale or clinical use. In practice, different food tables have been released that report the energy, macro-, and micronutrient contents of each food. Individual habitual EI can be assessed using longitudinal or retrospective methods, all of which are prone to reporting errors [21]. Longitudinal methods for evaluating EI, such as food diaries, involve the use of foods and drinks introduced when consumed for at least three consecutive days; this approach enables both qualitative and quantitative evaluations of dietary habits. Retrospective methods primarily include dietary recalls and food frequency questionnaires (FFQs), which rely on the memory of foods consumed prior to the interview conducted by an experienced professional (i.e., a dietitian). These approaches are easier to administer than longitudinal methods but are limited by reliance on memory, which can affect accuracy. Additionally, they do not permit accurate bromatological analysis. The 24-h recall (24-HR) consists of asking the participants to recall everything they ate and drank in the previous 24 h. With adequate software, the contents of energy and macronutrients are subsequently computed. FFQs are inexpensive and easy-to-use instruments for assessing habitual food intake, especially in epidemiological research. The questionnaire includes a list of foods and beverages, and the participants are requested to indicate their habitual consumption over a specific period (e.g., one week). Because food consumption patterns vary across cultures and geographic areas, FFQs must be properly validated for the specific population being investigated. In addition, assessing the accuracy with which the questionnaire measures the specific foods or nutrients that need to be detected is essential [22]. An important issue is that people with obesity are known to underreport, in most cases subconsciously, their intake of food, thus limiting the accurate estimation of EB [23, 24].

## Energy balance regulation: role of the ponderostat

**Fig. 3:**
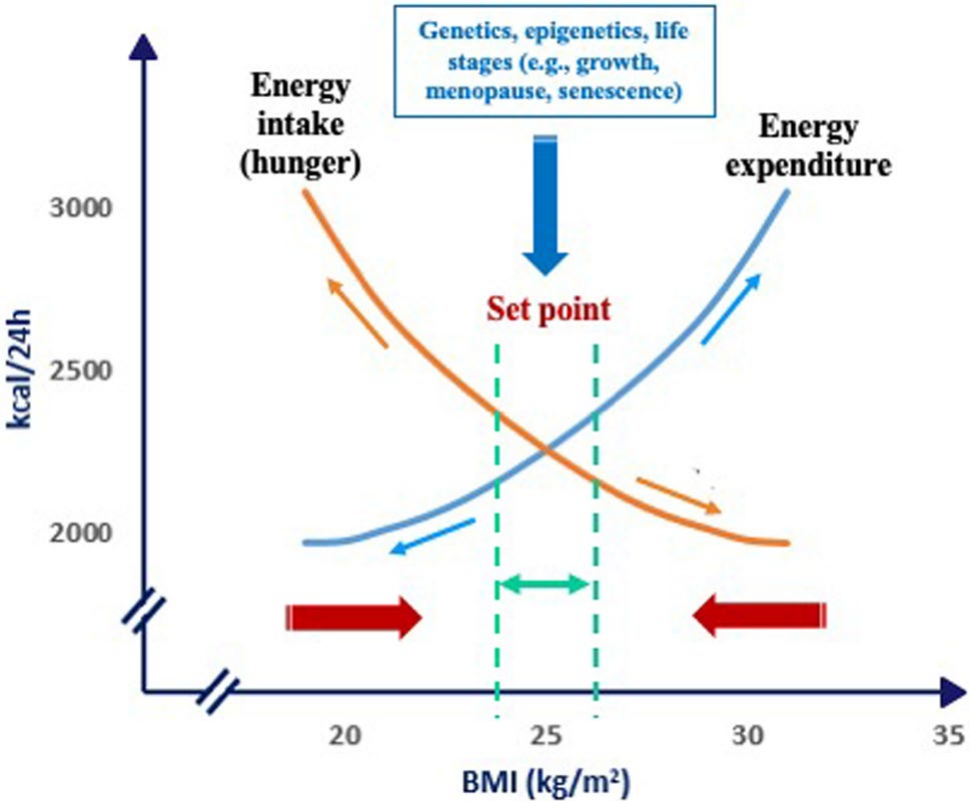
The theory of the set point

Paradoxically, a strict application of the energy balance (EB) equation might lead to misinterpretation. In fact, if we hypothesize that the daily intake of a slice of bread exceeds the daily energy requirements, it would lead to a weight gain of 20 kg in 10 years. However, this contradicts all evidence in clinical practice, and we must admit the existence of regulatory mechanisms that can mitigate the effect on body weight of any variation in excess or deficit of both energy intake and expenditure [24, 25]. Body weight is regulated within an individual’s range conditioned by genetic and/or epigenetic factors that can also vary at different stages of life: growth, aging, menopause, pregnancy, and lactation [26]. This body weight range can be referred to as the *set point*. Recent research seems to agree with the concept of the set point, demonstrating that adipose tissue retains an epigenetic memory that, following body weight reduction, activates metabolic changes that act against body weight changes [27]. Therefore, in the presence of factors involving the EI or the EE, the body activates responses that modify the EB to maintain a body weight that is stable in the range of the set point (Fig. 3). A link between EE and food intake was previously proposed, hypothesizing that EE is the primary determinant of food intake [28]. This hypothesis was based on the assumption that EE, reflecting the body's energy needs, may regulate food intake by centrally modulating hunger. This regulation would be ensured by *energy-sensing* mechanisms that include hormonal pathways or body compartments, or both. Therefore, a relatively high EE may increase hunger and food-seeking behavior in most individuals, perhaps even leading to higher-than-needed energy intake in some cases and, ultimately, weight gain. The set point theory has the merit of considering body weight as the primary determinant of energy balance, explaining several situations that, in some cases, may appear contradictory when considered according to the energy-sensing theory. For example, if an overweight individual decided to improve lifestyle by increasing physical activity, the consequence would also be an increased energy intake, which is contrary to actual experience in most cases.

The adaptive variations in energy balance in response to changes in body weight are described (when body weight reduces or increases beyond the set point, opposite effects (red arrows) on the energy balance take place to restore body weight).

**Fig. 4:**
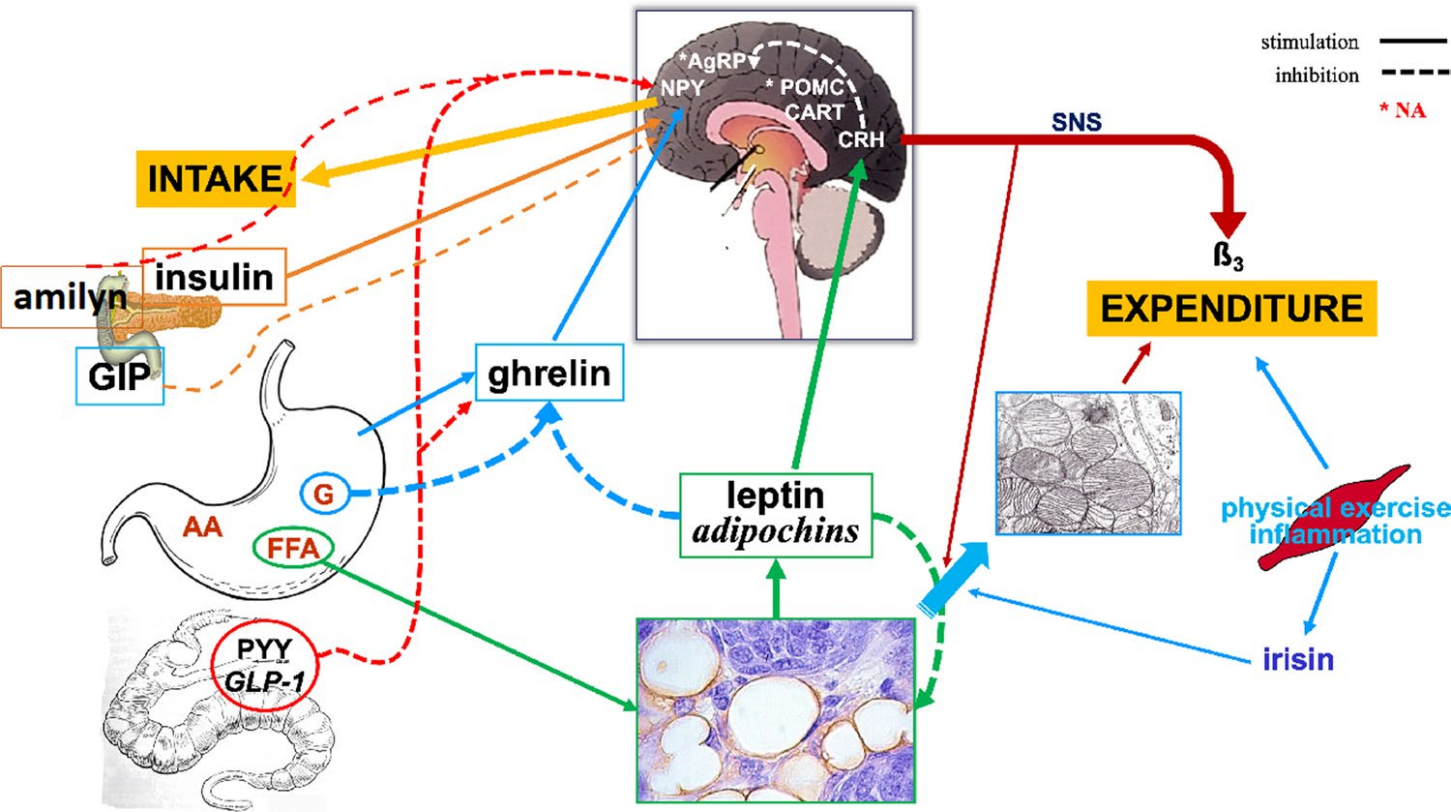
Neuroendocrine regulation of energy balance and the ponderostat

When body weight increases (positive EB) above the range of the *set point*, mechanisms leading to a reduction in EI and an increase in EE are activated. Conversely, when body weight decreases (negative EB), as in the case of starvation or weight loss diets, EE decreases, while hunger and thus food seeking and consumption increase, thus promoting an increase in EI. This is considered a fundamental survival mechanism of the human species, particularly in the context of food shortages or increased energy demands [25]. The concept of the body weight set point suggests that each individual has an “ideal” or “preferred” body weight that the ponderostat strives to maintain. The body weight set point is likely not a fixed, unchanging value but rather a range of body weights that can be influenced by genetic, environmental, and behavioral factors [29]. The concept of a body weight “set point” suggests that each individual has a biologically regulated range of body weights actively defended by neuroendocrine mechanisms. However, the rigidity or plasticity of this set point appears to vary between individuals. The evidence supporting a fixed set point includes the well-documented difficulty that many individuals with obesity face in achieving and maintaining weight loss. This resistance suggests that, in some cases, the ponderostat may be calibrated to a higher body weight level, actively opposing efforts to reduce it. Prolonged caloric restriction often elicits compensatory physiological responses, such as reductions in the basal metabolic rate and increases in appetite, which hinder long-term weight maintenance. In this context, obesity might be considered a disease of the ponderostat. The ponderostat orchestrates these compensatory responses and is considered a dynamic neuroendocrine network that continuously monitors energy status, integrating central and peripheral signals to ensure energy homeostasis and maintain a relatively stable body weight over time (Fig. 4).

**Fig. 5:**
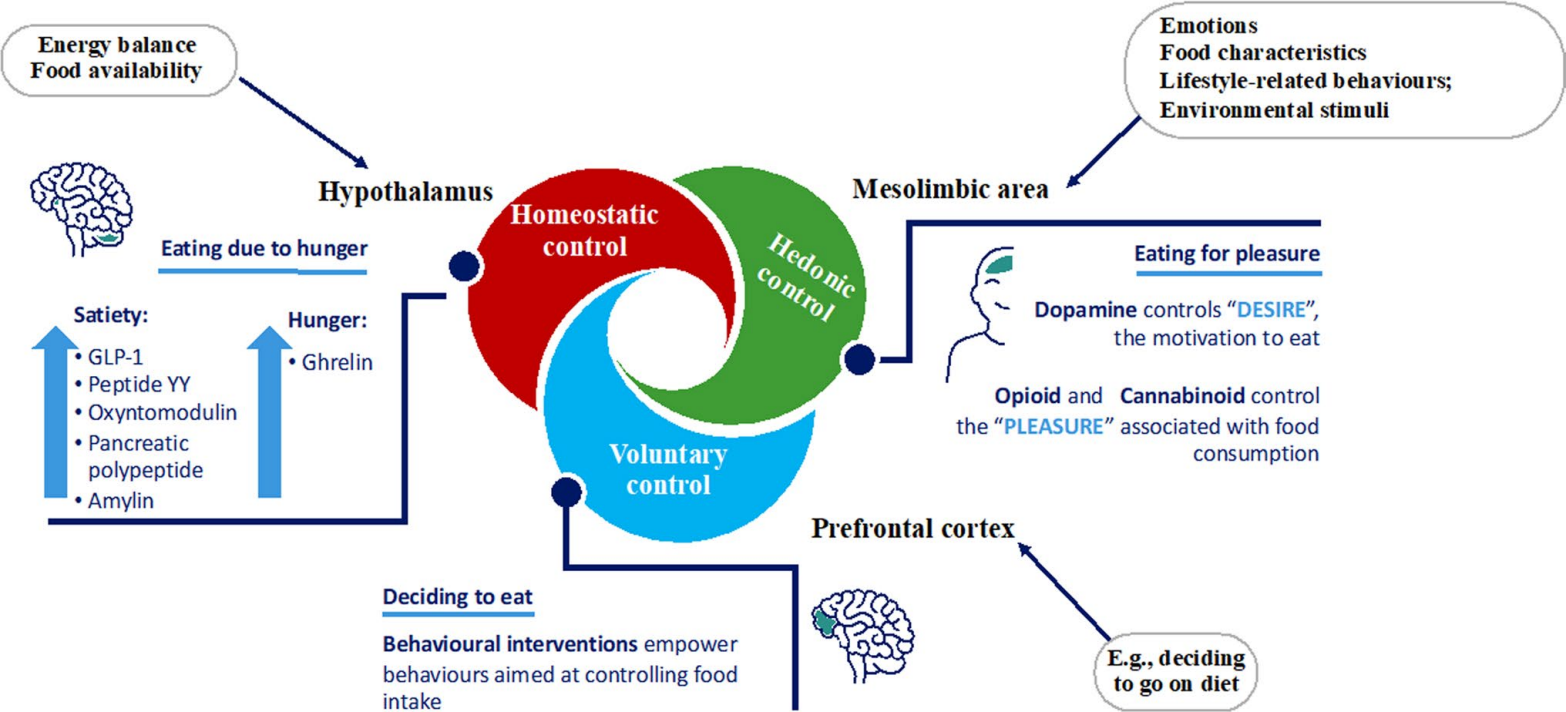
Role of the brain in the regulation of food intake: ponderostat

Receptors in the mouth, stomach, and bowel detect the taste, smell, texture, and volume of food, sending signals to the brain that influence appetite and satiety. Blood glucose, amino acids, and fatty acids provide direct information about the body's energy status to the brain. To the best of our knowledge, the ponderostat is organized such that the hypothalamus acts as a central hub, integrating peripheral inputs and modulating the neuroendocrine response [29–33]. Specific key hypothalamic nuclei are involved and include the arcuate nucleus (ARC), paraventricular nucleus (PVN), dorsomedial nucleus (DMN), and lateral hypothalamic area (LHA). Within the ARC, two antagonistic neuronal populations regulate energy balance: POMC/CART (pro-opiomelanocortin/cocaine- and amphetamine-regulated transcript) neurons, which suppress appetite and enhance EE, and NPY/AgRP (neuropeptide Y/Agouti-related protein) neurons, which stimulate appetite. POMC is a precursor of several peptides, including α-MSH (α-melanocyte-stimulating hormone), which activates melanocortin receptors (MC3R and MC4R) and promotes satiety; similar actions are carried out by CART, which suppresses appetite. Conversely, AgRP acts as an antagonist of these receptors, blocking MSH activity and promoting feeding behavior. NPY is among the most potent orexigenic peptides, enhancing food intake and fat storage and reducing EE. The PVN receives input from the ARC and regulates autonomic and endocrine responses by releasing corticotropin-releasing hormone (CRH) and thyroid-stimulating hormone (TSH), influencing appetite and EE. CRH suppresses appetite and increases EE in response to stress, acting on β3 adrenergic receptors. The LHA contributes to food-seeking behavior and energy intake via orexigenic peptides such as orexin and melanin-concentrating hormone (MCH). Orexin stimulates arousal, appetite, and physical activity, whereas MCH promotes feeding and reduces EE. Peripheral hormones provide essential inputs to the central nervous system in terms of energy stores and recent nutrient intake. Leptin, which is secreted by white adipose tissue (WAT), is a long-term signal of the body's energy reserves. It suppresses appetite, crosses the brain barrier, acts on the hypothalamus, and, according to animal studies, increases EE via its action on hypothalamic neurons (Fig. 4). Circulating leptin concentrations in humans, correlate strongly with body fat percentage, are elevated in obesity and reduced in leanness. In humans, fasting induces a drastic hypoleptinemia and increases appetite that may be prevented by leptin administration. Leptin administration, in obese individuals with leptin deficiency, induces significant weight loss. No weight loss following leptin administration is observed in obese individuals with no leptin deficiency, indicating a leptin resistance. Leptin resistance, which is a common trait in obesity, likely impairs the feedback between fat mass and hypothalamus, leading to dysregulated appetite and energy storage [32, 34]. Ghrelin is secreted primarily by P/D1 cells in the fundus of the stomach and acts as a short-term hunger signal, stimulating NPY/AgRP neurons in the ARC. Its levels increase before meals and decrease after meals [32]. Additional hormones, such as insulin and amylin, are cosecreted by pancreatic β-cells and modulate satiety. Insulin acts centrally to reduce food intake, whereas amylin slows gastric emptying and enhances postprandial satiety. Some gut-derived peptides play a critical role in short-term appetite regulation. After food intake, the ileum and colon secrete peptide YY (PYY), which contributes to the inhibition of appetite at the hypothalamic level. Glucagon-like peptide-1 (GLP-1), produced by L-cells in the ileum, promotes satiety, delays gastric emptying, and enhances insulin secretion; these mechanisms make GLP-1 a target for antiobesity and antidiabetic treatment [31]**.** Cholecystokinin (CCK), which is released in response to the intake of fats and proteins, also contributes to early meal termination, promoting satiety through vagal afferences. Glucose-dependent insulinotropic polypeptide (also known as gastric inhibitory peptide, GIP) is produced by the enteroendocrine K cells of the duodenum and upper jejunum following meal ingestion; similar to GLP-1, it has an insulinotropic effect and contributes to glucose homeostasis. GIP regulates appetite with an anorectic effect; however, the mechanisms of action are still not entirely known. Irisin is a hormone produced by skeletal muscle (myokine) in conjunction with physical exercise and has been identified as an essential mediator of browning [16]. In addition to the hypothalamic center, at least two other brain areas are critical for controlling energy balance and food intake and may be included as further structures of the ponderostat (Fig. 5). The hypothalamic portion of the ponderostat is responsible for the homeostatic control of hunger, satiety, and energy expenditure. That is, the regulatory functionality that enables the body to maintain stable internal conditions despite changes to maintain conditions suitable for survival. The prefrontal cortex is responsible for the voluntary control of hunger and contributes to cognitive restraint and executive control over eating behavior. It can influence appetite and food preferences in response to stress, rewards, and memories associated with food. A third important area of the ponderostat is the limbic system (amygdala, hippocampus, and cingulate cortex), which modulates emotional and reward-driven eating (cannabinoids and dopamine) [33]. The prefrontal cortex will decide to embark on a dietary pathway, but, sometime later, it must deal with the hypothalamic and mesolimbic areas, which control appetite in a homeostatic or hedonic manner. Unfortunately, very often, the prefrontal cortex has to succumb to the intervention of these other areas, possibly preventing the continuation of the dietary course or returning to the starting weight condition, if not to further weight gain. Understanding the regulation of the ponderostat and its disruption is crucial for developing effective weight management strategies for obesity.

## Increased energy efficiency and obesity

Compared with nonobese individuals, individuals with obesity are characterized by a higher EE. Therefore, obese individuals must intake a greater number of calories for a sufficiently prolonged period of time than nonobese individuals to achieve and maintain the condition of obesity. As previously reported, a sufficiently protracted period of positive EB induces the ponderostat to increase the EE, namely, DIT. However, it is possible that some individuals are more metabolically efficient than others are, possibly because their ponderostat is regulated toward an energy-sparing state. Therefore, a low relative energy expenditure may facilitate the development of obesity. Once obesity is established, energy dissipating mechanisms are activated by the ponderostat such that identifying a low relative energy expenditure is hardly possible, especially if obesity is defined only on the basis of anthropometric measurements and not discriminating individuals on the basis of clinical and pathological factors, mixing together metabolically sparing and nonsparing persons. These considerations may explain why an energy-sparing condition is more easily demonstrated in preobese people than in individuals with established obesity. The data presented in Fig. 2 demonstrate that the RMR may vary within a range of approximately 500 kcal between people with the same FFM, namely, the most important factor influencing the RMR [35]. Therefore, we can hypothesize that those people who are more metabolically efficient can more easily reach a positive EB and a condition of obesity than individuals with a higher RMR can. These people may be at risk of obesity even with small extra increases in usual caloric intake. A positive EB leads to an increase in body weight, which also includes an increase in FFM, which translates to an increase in the RMR. Furthermore, as previously observed, under conditions of a positive EB, the ponderostat is activated to increase the EE (DIT) and limit body weight gain. Finally, a rebalancing of the EB is obtained at a higher body weight with a lower possibility of demonstrating an energy-saving state at this stage.

**Fig. 6:**
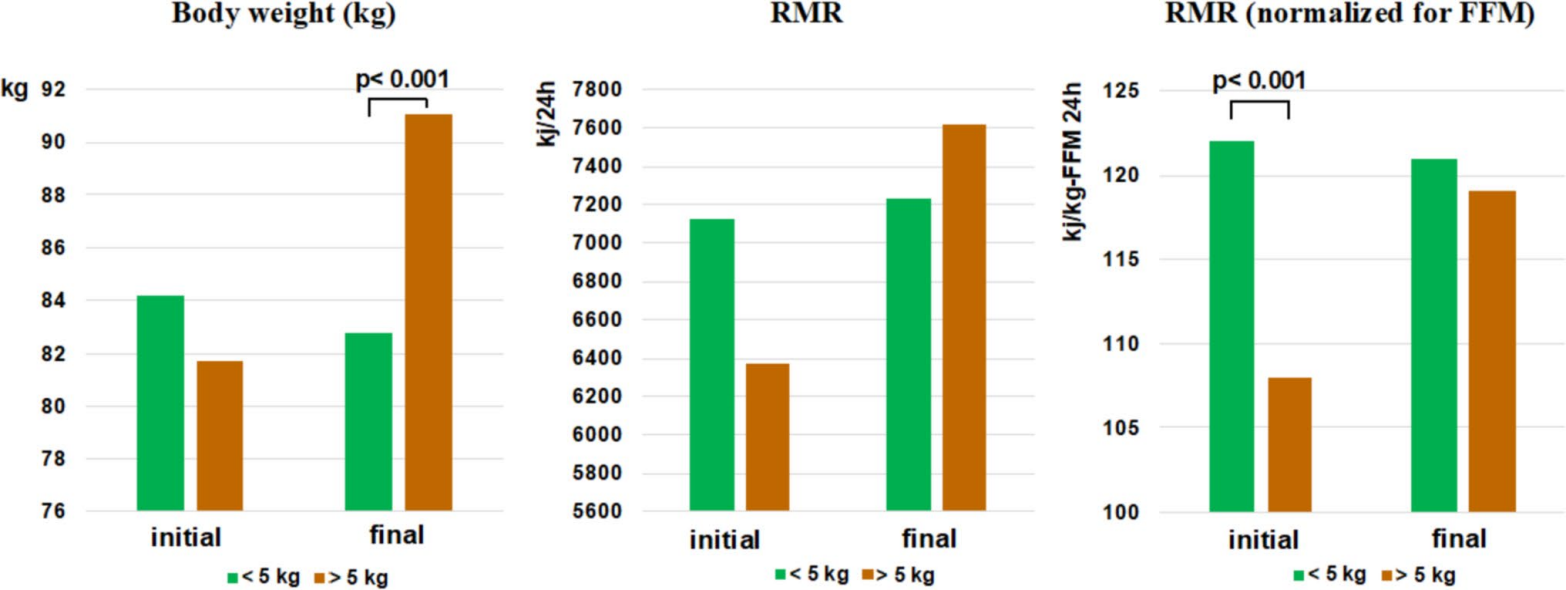
A low relative resting metabolic rate is associated with significant body weight gain in preobese individuals (adapted from Ref. [40])

**Fig. 7:**
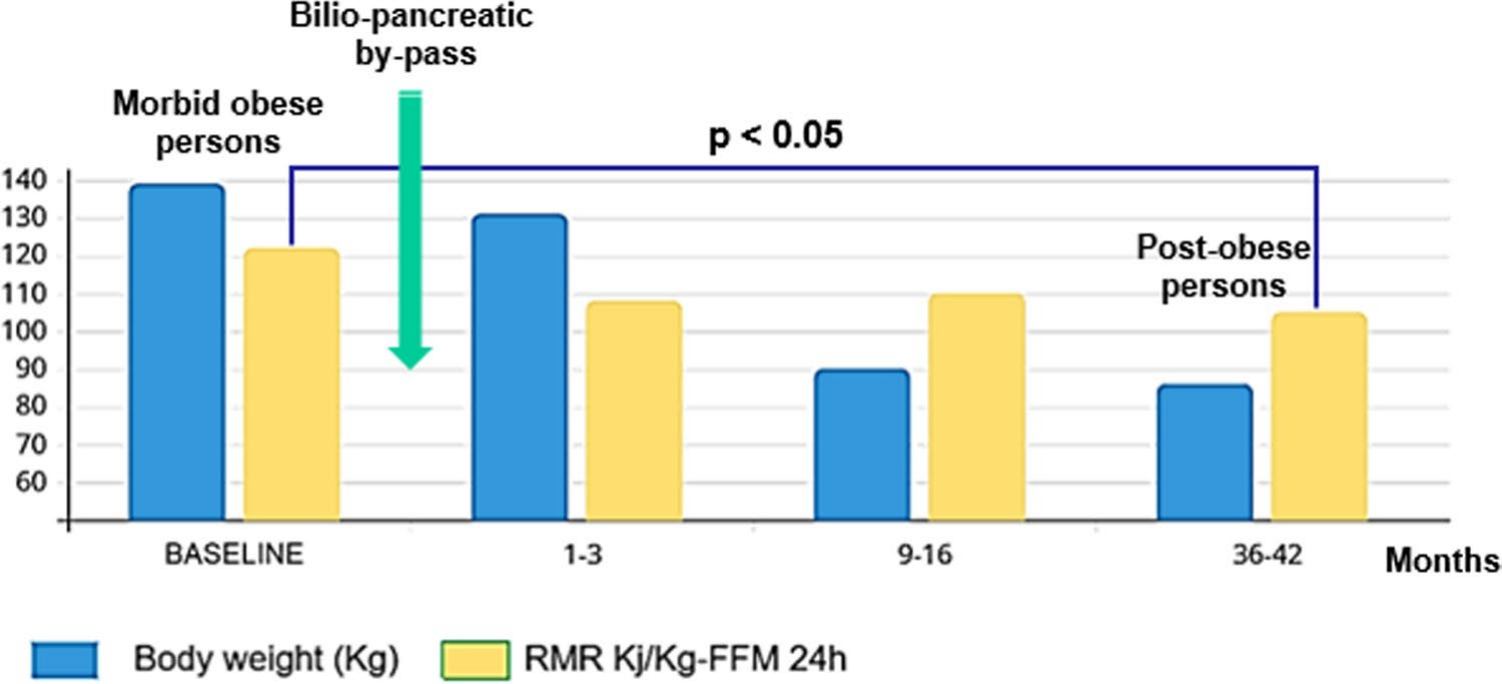
Postobese individuals following bariatric surgery exhibit a low relative energy expenditure (RMR) normalized for fat-free mass (FFM) (adapted from Ref. [41])

Different studies have demonstrated longitudinally that a low relative EE is predictive of future weight gain. Ravussin et al. observed Pima Indians over a follow-up of 2 years and found that a low relative 24-h energy expenditure, measured with a metabolic chamber, or a low relative RMR, measured with indirect calorimetry, were associated with body weight gain [36]. In infants born to obese mothers, Roberts et al. observed that a low 24-h EE at birth was associated with greater body weight gain at 9 months of age than in infants of normal-weight mothers [37]. Griffith et al. demonstrated longitudinally, with a follow-up of 12 years, that in 5-year-old girls, a low energy expenditure was predictive of greater body weight gain [38]. Longitudinal studies on adult Caucasian people are contradictory. Seidell et al. reported that a low relative RMR was not associated with future body weight gain; however, many methodological limitations (age range of included adults of 19–98 years old, use of an old and inaccurate method to measure energy expenditure) make these conclusions questionable [39]. Another longitudinal study from our group with a follow-up of 8–12 years confirmed in adult Caucasian persons that a low relative RMR was predictive of weight gain (Fig. 6) [40]. Therefore, the possibility exists of identifying preobese individuals who have an energy-sparing metabolism, which might allow targeted actions to prevent the evolution toward obesity in biologically predisposed individuals. In addition to studies on preobese people, another strategy is to investigate stable postobese people as a result of bariatric surgical treatment. We observed [41] that, 36–42 months after bariatric surgery, when a stable postobese condition was obtained with an average weight loss of approximately 55 kg, the RMR normalized for FFM was significantly lower than that before surgery, thus suggesting the presence of increased energy efficiency (Fig. 7). In some instances, an energy-sparing condition may result in reduced postprandial thermogenesis, namely, the ability to dissipate some ingested calories as heat. Individuals with obesity and a strong family history of obesity-diabetes exhibited lower PPT than equally obese people but without a family history of obesity-diabetes [42]. Some pharmacologically active substances can increase or reduce EE. Sympathomimetics, theophylline, salbutamol, and thyroxine (at supraphysiological doses), or substances such as nicotine have recognized thermogenic actions [43], and this property partly explains (there is a concomitant compensatory phenomena that increases appetite) why stopping smoking is almost invariably followed by weight gain [44]. Conversely, beta-blockers reduce EE [17]. Several studies have documented how beta-blocker treatment is associated with an increase in body weight estimated at approximately a median value of 1.2 kg but up to a maximum of 3.5 kg; this effect is attributable to a reduction in EE (RMR, postprandial thermogenesis, possible reduction in adipose tissue browning) [45]. Another possible energy-sparing disorder is the weight-cycling (WC) condition, which identifies people with obesity who reduce their body weight and subsequently regain it. With each weight cycle, they are often less able to reduce their body weight and subsequently regain it to a greater extent than they have lost. WC might be favored by an energy-saving condition because of the poor adaptive capacity of the ponderostat such that the EB can more easily become positive again and increase body weight. We previously presented [46] a case report of a person with WC and demonstrated on two different occasions (with approximately the same body weight and after weight regain) that the RMR, expressed in absolute value or normalized for FFM, was significantly reduced in the second occasion, thus suggesting an energy-sparing condition. Therefore, the WC phenomenon might favor and be favored by increased energy efficiency; however, the condition of WC remains poorly defined and underinvestigated.

**Fig. 8:**
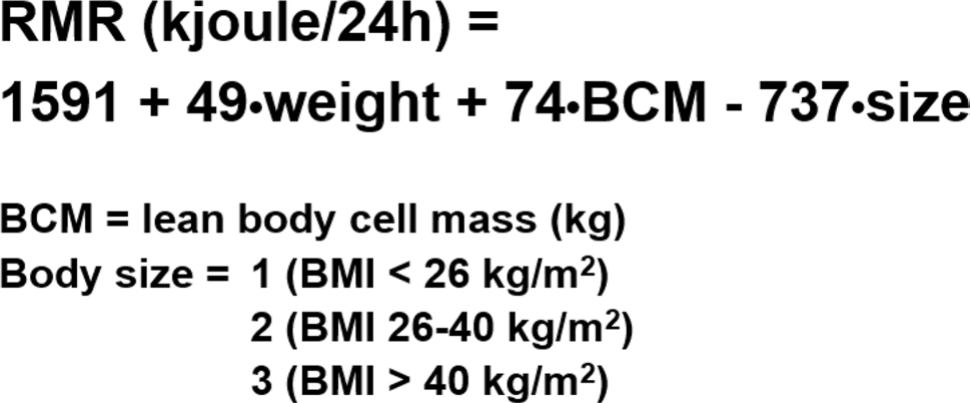
Predictive equation of the resting metabolic rate obtained from a multivariate analysis of a large database (adapted from ref. [42])

Metabolic syndrome is likely another clinical condition associated with energy-sparing metabolism and obesity. We observed that compared with nonobese or obese individuals without metabolic syndrome, obese individuals with metabolic syndrome had a significantly lower normalized RMR for FFM [47]. In the same study, it was observed that when metabolic syndrome evolves in diabetes, it is associated with normal RMRs. This effect is likely due to the increase in the neoglucogenesis activity, an energetically expensive metabolic pathway, which occurs at a relatively high rate in the context of diabetes [48]. A bolus of fast-acting insulin in people with type 2 diabetes and fasting hyperglycemia significantly reduces the RMR, probably because of the inhibition of neoglucogenesis activity, as seems to be indicated by a concomitant reduction in blood glucose and lactate concentrations [49]. These data may help explain why insulin treatment in people with diabetes is associated with weight gain and positive energy balance [50]. In fact, peripheral hyperinsulinization induces appetite and reduces glycosuria, therefore increasing energy intake; on the other hand, it reduces energy expenditure by reducing neoglucogenesis activity. An analysis of the predictive equation for the RMR presented in Fig. 8 and data obtained from a large database revealed greater energy efficiency with increasing body size. In fact, for the same body weight and body cell mass (BCM), two individuals with different body sizes (e.g., hypothesizing different heights) would have different RMRs; in particular, the RMR would be lower for the individual with a greater body size [42]**.** This observation is in agreement with the existence of a biological predisposition that favors positive EB mediated by a low relative EE. An impaired browning ability has emerged as a factor that may be associated with an energy-saving condition related to the development of obesity [51]. Genetics plays a significant predisposing role for obesity, and twin and family studies have shown that body weight is a highly heritable (40–70%) trait [52]. Several obesity-related genes that influence appetite, EE, nutrient metabolism, and body fat distribution have been identified. Fat mass and obesity-associated gene (FTO) variants have been found to be associated with obesity. For years, it was unclear how variants in FTO influenced body weight. In 2015, Claussnitzer et al. uncovered the biological mechanism behind this association [53]. They found that a noncoding variant within the FTO locus alters the function of a regulatory element. This variant affects the expression of distant genes, particularly IRX3 and IRX5, in preadipocyte cells (precursors of fat cells). Individuals with the obesity risk variant show a shift in energy balance, favoring white fat cell development (which stores energy) over beige fat cell development (which burns energy). This leads to reduced thermogenesis and increased fat accumulation, ultimately promoting obesity. This study was a major breakthrough, showing that FTO affects obesity not by acting directly but by regulating other genes involved in fat cell biology.

## Challenges related to appropriate analysis of energy intake

Energy intake as a component of energy balance probably needs to be reconsidered when understanding obesity. An inevitable incorrect estimation of caloric intake, genetic and epigenetic factors, and the microbiota may be some of the factors determining different responses in terms of body weight change for the same EI. An erroneous estimate of the energy content of foods compared with what is reported in food tables and databases cannot be ruled out. In particular, the energy content of foods may vary for the same food depending on its method and place of production. The bromatological analysis of food is performed through specific laboratory methods. For instance, water content is determined by weighing food before and after drying, while protein is calculated through the Kjeldahl method, which measures total nitrogen and converts it into protein. Fats are extracted with solvents such as ether, ash is obtained by burning the organic part in a high-temperature oven, and carbohydrates are estimated by the difference, that is, by subtracting the sum of the other nutrients from the total mass. This is not a perfect system, and miscalculations can occur. One of the most common errors concerns the calculation of carbohydrates, which are not measured directly but are obtained by the difference: the sum of water, protein, fat, ash, and fiber is subtracted from the total mass. This means that any inaccuracies in the analysis of individual components are invariably reflected in the final estimation of the amount of carbohydrates. The protein content, which is calculated by the Kjeldahl method, may also be overestimated because it measures total nitrogen, including nonprotein nitrogen compounds (such as nitrate or urea). Other errors may result from losses of volatiles during drying or incineration, instrumental inaccuracies, or natural variability in the feed. In addition to analytical aspects, processing conditions and cooking methods can also profoundly affect the nutritional profile of food. Furthermore, adding ingredients such as sugars, fats, or sodium during processing changes the original nutritional content, often worsening it [54]. Home cooking may also have a major effect. Methods such as frying can increase the fat content and consequently the energy content. In contrast, gentler cooking methods such as steaming or microwaving tend to better preserve the nutritional value of foods. Therefore, significant differences are expected to occur between the estimation of EI using databases and what is actually introduced. Furthermore, this estimation is necessarily an approximation since we consider foods with the same characteristics as similar foods indicated by databases as the same foods, which may have different characteristics because of heterogeneous production procedures. Another important point is that, probably, we erroneously assume that all the energy introduced with food is entirely utilized. In a recent study, we observed in a representative sample of the local population that a polymorphism of the gene coding for PNPLA3 (patatin-like phospholipase domain containing 3), known to be associated with hepatic steatosis, was also independently associated with body weight gain [55]. In particular, those persons who gained body weight over a 4-year time interval, in addition to having increased their habitual EI, exhibited a higher prevalence of the CG and GG alleles of the PNPLA3 gene. This gene is involved in mechanisms of autophagy that allow the destruction and elimination of a significant amount of fatty acids when excessive influx to the liver occurs, such as during overfeeding. It would therefore be possible to eliminate with bile salt part of the lipid energy substrate introduced with food, reducing its effect on energy balance. It is hence hypothesized that where this gene is functionally less efficient, it may promote obesity (as well as steatosis). According to the results of the theoretical calculations, it was plausible that some of the observed weight gain following overeating could be traced to less efficient autophagy mechanisms. In some instances, the concomitance of all these factors may, at least in part, explain what some people report about not eating as much as expected for their excessive body weight. Another possible confounding factor when effective energy intake is considered is fiber intake. For many years, it was assumed that one could consume “unlimited” amounts of fiber without influencing the EI and the EB because they are not absorbed by the intestinal wall. However, some of the fibers introduced with the diet are processed by the intestinal microbiota, absorbed and metabolized. In particular, the gut microbiota metabolizes part of the dietary fiber, producing short-chain fatty acids (SCFAs, mainly acetic, propionic, and butyric acids), which are subsequently absorbed and ultimately used as a source of energy, thus escaping the count of calories introduced with the diet [56].

Finally, even the timing of eating may result in a positive energy balance and increased obesity risk [57]. In particular, late dinner timing increases obesity risk by disrupting circadian and neuroendocrine regulation of appetite and energy expenditure, especially in the context of POMC, ACTH, and cortisol dysregulation, and this effect is exacerbated by other pituitary or hypothalamic dysfunctions [58–60]. In a recent controlled crossover trial, late eating led to higher subjective hunger, an increased ghrelin:leptin ratio, lower waking energy expenditure, and reduced 24-h core body temperature, independent of total caloric intake or macronutrient composition [61].

## Pharmacological treatment of obesity and its influence on EB

Antiobesity medications exert their effects by altering energy balance. These antiobesity drugs can be broadly categorized into those that reduce EI, which are actually available on the market, and those that also modify the EE, which are currently under investigation.

### Medications that act on energy intake

#### Naltrexone/bupropion

This combination therapy includes naltrexone, an opioid receptor antagonist also used to treat alcohol and opioid dependence, which reduces the reward and craving associated with food consumption. Bupropion, a norepinephrine and dopamine reuptake inhibitor indicated for depression and smoking cessation, enhances hypothalamic pro-opiomelanocortin (POMC) neuron activity. This combination works synergistically by stimulating POMC neurons (via bupropion) while inhibiting their opioid-mediated autoinhibition (via naltrexone), leading to reduced appetite [62]. This treatment also seems to have a certain efficacy in patients with binge-eating disorder [63].

#### GLP-1 receptor agonists

Initially developed for type 2 diabetes management because of their insulinotropic effects, GLP-1 receptor agonists (GLP-1 RAs), such as liraglutide and semaglutide, have since demonstrated efficacy in obesity treatment [64]. They act on the hypothalamic ponderostat, reducing energy intake through suppressing appetite and enhancing satiety. Evidence suggests that these agents also influence food preferences and behavioral patterns, decreasing the appeal of high-calorie and high-fat foods, reducing cravings, and enhancing cortical control over food-related decisions, effectively mimicking behavioral therapy [65, 66]. Recently, semaglutide was coadministered with cagrilintide (CagriSema), an analog of amylin, a peptide hormone that is cosecreted with insulin and contributes to the regulation of body weight by reducing energy intake, reducing appetite and increasing satiety. CagriSema resulted in greater weight loss than cagrilintide or semaglutide used alone [67].

#### Dual GLP-1/GIP receptor agonist

Tirzepatide is currently the only approved dual GLP-1/GIP (glucose-dependent insulinotropic polypeptide receptor) agonist for the treatment of obesity. Additionally, this molecule acts on hypothalamic ponderostat targets to regulate food intake, leveraging the distinct expression profiles of GLP-1 and GIP receptors in subcortical brain regions. Unlike GLP-1 receptors, GIP receptors are functionally expressed in adipocytes, allowing direct adipocyte modulation. This dual agonism appears to enhance weight loss more effectively than single agonism in both preclinical and clinical models. It also contributes to sustained weight reduction by addressing multiple regulatory mechanisms of energy balance [68].

### Antiobesity drugs affecting both EI and EE

#### Retatrutide

Retatrutide is a triple agonist that targets receptors for GIP, GLP-1, and glucagon. While glucagon is traditionally recognized for its hyperglycemic counterregulatory effects, it also promotes energy expenditure, delays gastric emptying, reduces food intake, and stimulates lipolysis [69]. Preclinical studies have shown that triple agonists outperform dual GLP-1/GIP agonists in reducing body weight and enhancing glycemic control. Human trials suggest promising outcomes, with this novel class potentially offering superior efficacy in weight and metabolic management, probably also for an adjunctive effect on energy expenditure [70].

#### Survodutide

Survodutide is a dual agonist of glucagon and GLP-1 receptors. Preclinical models have shown that compared with semaglutide, survodutide induces greater weight loss at its maximum effective dose because of the synergistic activation of both the glucagon pathway and the GLP-1 pathway [71]. Its efficacy is partially attributed to increased energy expenditure through hepatic and adipose glucagon receptor activation and the stimulation of gluconeogenesis, glycogenolysis, and lipolysis. Currently under clinical investigation, survodutide represents a promising new avenue for obesity management, targeting both caloric intake and energy expenditure [72].

### Potential role of GLP1-RAs and dual agonists in “adiposopathy”

The term “adiposopathy” is not widely recognized in the literature. Adiposopathy is a condition driven by inflammatory mechanisms that prevent adipose tissue (mainly the subcutaneous compartment) from expanding in response to overfeeding, leading to the ectopic deposition of substrates, particularly lipids, as well as the release of inflammatory cytokines. Studies in experimental animals have also shown that one aspect of adiposopathy is the impaired ability to transdifferentiate ("browning") white fat into brown fat, an event that contributes to obesity [73, 74]. If we define adiposopathy as a disorder characterized by abnormal adipose tissue function and distribution, it could be considered a peripheral manifestation of dysregulated energy balance and metabolic control by the ponderostat. Adipose tissue dysfunction represents a major factor in the etiology of obesity and related complications since it promotes chronic inflammation, dysregulated glucose homeostasis, and compromised adipogenesis, leading to the accumulation of ectopic fat and insulin resistance [75]. In line with these concepts, both GLP-1RAs and dual GLP-1/GIP receptor agonists could represent pharmacological strategies capable of modulating adipose tissue metabolism, and although GLP-1 receptors are not expressed in adipose tissue, GLP-1RAs may influence fat metabolism through indirect mechanisms [76]. First, they increase lipolysis and fatty acid oxidation, increasing the breakdown of stored fat and promoting the utilization of fatty acids for energy purposes; second, they induce the “browning” of white adipose tissue, leading to increased energy expenditure; and finally, they increase the levels of adiponectin, an adipokine associated with improved insulin sensitivity and anti-inflammatory effects.

## Conclusions

We have information to support the unifying perspective that considers obesity as a biological condition with an abnormal set point and dysregulated energy balance due to abnormalities in ponderostat function. This hypothesis is consistent with the concept that obesity is a disease. Current and future antiobesity pharmacological treatments may be considered curative for ponderostat dysregulation and potentially more targeted. Furthermore, this perspective may contribute to counteract the prejudice, discrimination, and negative stereotypes that often characterize the obesity stigma. However, further studies are needed to provide more adequate evidence to support more appropriate diagnoses and treatments.

## Data Availability

No datasets were generated or analyzed during the current study.
